# Change in skin properties over the first 10 years of life: a cross-sectional study

**DOI:** 10.1007/s00403-017-1764-x

**Published:** 2017-07-19

**Authors:** Fanqi Kong, Carlos Galzote, Yuanyuan Duan

**Affiliations:** 1 Johnson & Johnson AP Skin Testing Center, Shanghai, China; 2 Johnson & Johnson International (Singapore) Pte. Ltd, 2 International Business Park, Singapore, 609930 Singapore; 3 AP Clinical Operations, Johnson & Johnson China Ltd., 3285 Dongchuan Road, Minhang, Shanghai, China

**Keywords:** Skin barrier, Skin condition, Transepidermal water loss, Water handling, Children, Adult

## Abstract

This study investigated skin characteristics in healthy Chinese children aged from 1 to 10 years and compared these findings with similar measures from the child’s mothers. Children aged 1, 2, 3, 4, 5, and 10 years (*n* = 29–30 per age group) and the child’s mothers were enrolled in a single-visit cross-sectional study. Clinical parameters evaluated on the face, ventral forearm, and calf were softness, smoothness, erythema, edema, rash, dryness, and scaling. Instrumental evaluations included transepidermal water loss, moisture content, and water-holding capacity. The clinical evaluations indicated a general decrease in softness, smoothness, and overall skin condition with increased child age. In general, the child’s clinical scores were better than in adults. Children had a more permeable skin barrier that matured to adult values by approximately 5 years of age. Mothers had greater skin moisture than children. Clinical and instrumental measures were consistent with skin being softer and smoother and in better overall condition in younger children. As the skin matured with age, higher scores were observed. Instrumental measures demonstrated a more permeable skin barrier in younger children compared with older children and with adults.

## Introduction

The skin of infants and children is often characterized as smoother and softer than in the adult [8]. In infants, the stratum corneum is thinner, water handling is different, and natural moisturizing factor and skin lipid production are reduced compared with adults [4–6, 11]. Because of these developmental differences, the skin in children may be more sensitive to irritation and inflammation [7]. These differences can help explain some of the increased sensitivity of the infant to specific skin insults that are not as prevalent in adults and that can result in diaper dermatitis and atopic dermatitis [2].

The goals of this study were to investigate the changes in skin and skin barrier function in children aged from 1 to 10 years and to compare these findings with similar measures (clinical dermatologic characteristics as well as instrumental and physical characteristics) from the child’s mothers.

## Methods

### Study design

This was a single-visit cross-sectional study in children of different ages (aged 1–5 and 10 years) performed from December 2012 to January 2013 in Shanghai, China. The mother of each child signed an informed consent form. The study was approved by an ethics committee or institutional review board and conformed to the latest revision of the Declaration of Helsinki and good clinical practice. Subjects were ethically compensated immediately after successful completion of the study in the form of small tokens, food, and/or a transportation allowance.

Assessments were made in six groups of healthy children aged 1, 2, 3, 4, 5, and 10 years and in each child’s mother. Skin properties as described below were assessed on the face, ventral forearm, and/or calf.

Inclusion and exclusion criteria are described in Table 1. The study subjects (mothers, children) were instructed to wash only with water ≥12 h before the clinic visit; no skin care product was to be used for 24 h prior to the visit.

**Table 1:** Inclusion and exclusion criteria

Inclusion criteria	Exclusion criteria
The participating mother is the one who regularly cares for the child. The mother must ≥18 years and be willing and able to follow all study directions, accept skin examination of the study	Child/mother participant who exhibits or is known to have atopic dermatitis, eczema, or other inflammatory disease, or mild-to-severe diaper rash or skin diseases/conditions that in the opinion of the investigator may affect the evaluation of study product or place the child at undue risk
Participating mother must be willing to not bathe her child and herself at least 12 h prior to the clinic visit and to have clean skin at the clinic visit	Any children and mothers with clinically determined moderate-to-severe dryness or clinically determined erythema, rash, or other skin condition
Mother must be willing to not apply any skin care product to the child and herself for 24 h before visit until after the visit is completed	Any condition requiring use of a topical or oral OTC or prescription medication, which, in the investigator’s judgment, makes the subject ineligible or places the subject at risk. Child vitamins were allowed
	Individuals currently involved in another clinical study of any type
	Child participants who experienced moderate-to-severe diaper rash within 1 week prior to study start
	Child/mother participant who has experienced unusual hypersensitivity or allergic reactivity to fragrances and/or reaction/irritation to skin care toiletry products
	Child/mother participant with a known condition of asthma or any related breathing problems and/or for whom there is a family history of asthma
	Mother of the child is an employee of the investigator or study center or is a family member of the employees or the investigator

### Dermatological assessments

A single dermatologist evaluator performed all grading for all of the subjects in the study. The evaluated clinical parameters were overall skin condition, softness, smoothness, erythema (irritation), edema (irritation), rash (irritation), dryness, and scaling. Clinical assessments were made on the face, ventral forearm, and/or calf, and graded on a scale of 0 (none), 1–2 (very good), 3–4 (slight), 5–6 (medium or moderate), 7–8 (medium–severe), to 9 (severe). As described in Table 1, subjects with clinical scores in the moderate/medium-to-severe range were excluded from study entry.

### Instrumental evaluations

Transepidermal water loss (TEWL) measurements on the cheek were obtained using a VapoMeter (Delfin Technologies, Finland), which uses a closed-chamber measurement principle to evaluate water evaporation from the skin [1]. The probe was placed into contact with the skin surface, and one measurement was recorded.

Skin moisture content was measured using a Corneometer^®^ (CM 825, Courage + Khazaka electronic GmbH, Cologne, Germany) on the cheek and forearm (capacitance measure of surface skin moisture content, average of three readings per site) and a Skicon device (IBS Co. Hamamatsu, Japan), for high-frequency electric conductance measure of skin moisture content on the ventral forearm [9].

Water-holding capacity was tested using the Skicon device (IBS Co.) on the ventral forearm, as previously described [10]. After a baseline measurement was made, a drop of water was placed on the skin for 10 s and blotted. A measurement was then performed immediately thereafter (0 s) and at 30, 60, and 120 s. The entire test was repeated five times, with the average of three determinations being used to calculate water sorption and desorption (after removal of the minimum and maximum data points), according to the following formulas:

1 $$ {\text{Water sorption }}({\text{WS}}) = (G_{0} - G_{\text{BL}} )/ 30, $$

and

2 $$ {\text{Water desorption }}({\text{WD}}) = (G_{ 30} - G_{0} )/ 30. $$

### Data analysis

Dermatological assessments and instrumental assessments were summarized by mean ± standard deviation (SD), unless otherwise specified. The relationship of the skin evaluation data between babies and their mother was analyzed using Pearson correlation coefficient. The cross-sectional evaluation pertains to group comparisons of skin evaluation data of both babies and their mothers. It included comparisons of all age groups and pairwise comparisons among the age groups. Univariate cross-sectional comparisons (testing the difference among the age groups) were conducted using analysis of variance (ANOVA) and were assessed by non-parametric Kruskal–Wallis test. Differences between means were evaluated by the non-parametric Mann–Whitney *U* test or by the independent *t* test. The significance level for statistical tests of differences among the groups was set at 0.05. The Bonferroni method was used to account for cases of multiple comparisons.

## Results

In this study, 384 eligible subjects were recruited (192 children and 192 mothers) and 358 subjects completed the study. Demographics are shown in Table 2. There was an even distribution of boys and girls in the study and 30 children in each age group (except for 3 years with *n* = 29). The average age of mothers ranged from 29 ± 4 years for 1 year to 36 ± 7 years for 10 years.

**Table 2:** Participant demographics by children’s age

	1 year (*n* = 30)	2 years (*n* = 30)	3 years (*n* = 29)	4 years (*n* = 30)	5 years (*n* = 30)	10 years (*n* = 30)
Males, *n* (%)	16 (53%)	13 (43%)	15 (52%)	15 (50%)	16 (53%)	15 (50%)
Females, *n* (%)	14 (47%)	17 (57%)	14 (48%)	15 (50%)	14 (47%)	15 (50%)
Mother’s age, years (mean ± SD)	29 ± 4	29 ± 4	32 ± 9	32 ± 7	33 ± 6	36 ± 7

*SD* standard deviation

### Dermatological assessments

#### Assessments in children

**Fig. 1:**
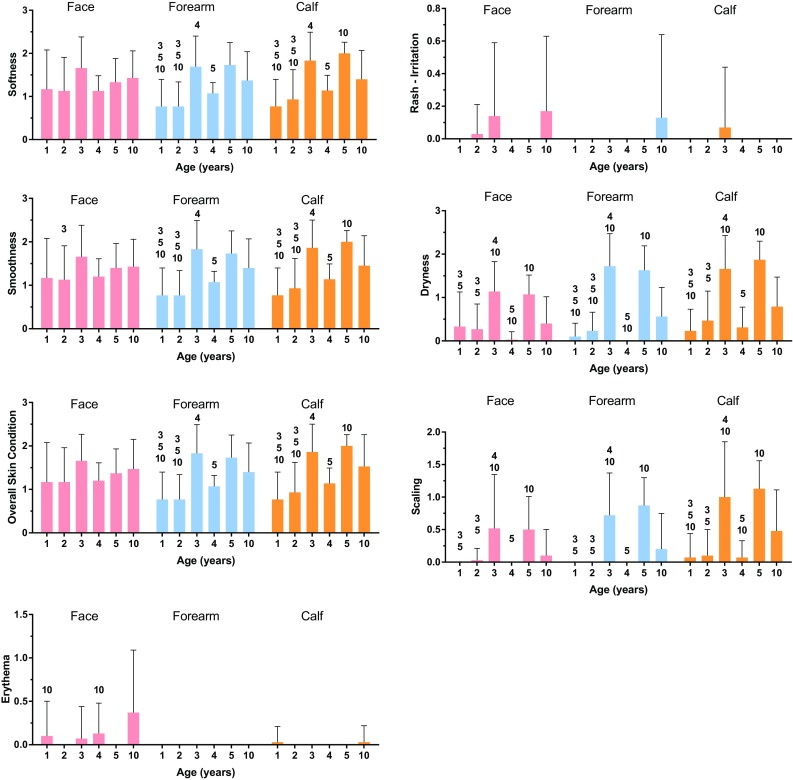
Dermatological assessments. A* number above* the* bar* in an age group indicate that a significant difference was between this age group and the other age groups identified

**Fig. 2:**
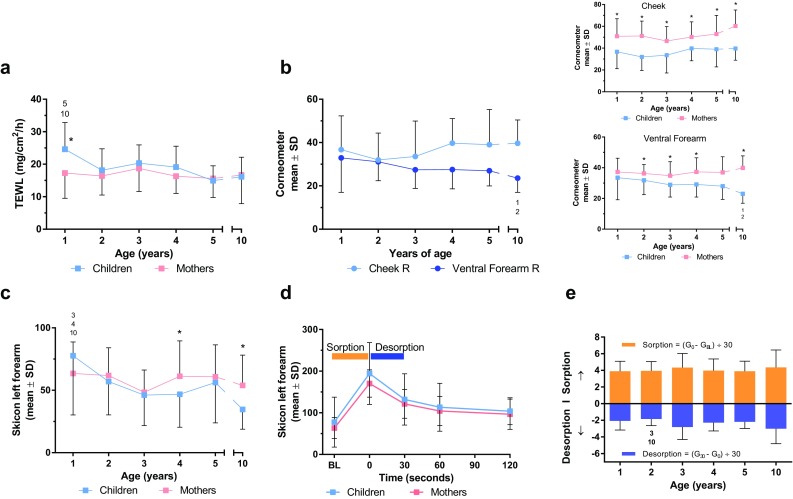
Measures of skin barrier function and skin moisture content or hydration **a** TEWL, **b** Corneometer (capacitance) in cheek and ventral forearm, **c** Skicon (conductance), **d** water-handling time course in 1 year, and **e** water-handling changes with age. A* number above* the* bar* or symbol in a children’s age group mean that a significant difference was observed between this age group (the *bar*) and the groups shown (*p* < 0.05). *p < 0.05 children versus mothers (*t* test)

In the assessments of the children’s facial skin, significant differences across age groups (*p* < 0.05) were observed for all dermatology variables (Fig. 1), except edema (not shown) when analyzed by ANOVA and Kruskal–Wallis test. In the pairwise age-group comparisons, there were generally no significant differences between age groups in mean scores for facial softness, smoothness, overall skin condition, and erythema, edema, or rash (measures of irritation) (Fig. 2). Differences among age groups were noted for dryness and scaling, possibly because dryness and scaling scores of 3- and 5 years were higher than in the other groups (Fig. 1). In spite of the fact that mean values for facial dryness and scaling in 3 and 5 years were higher than in the other groups, these values were all less than 2, which was still considered in the very good range.

In the assessments of the ventral forearm and calf, there were significant differences across groups for softness, smoothness, overall skin condition, dryness, and scaling (*p* < 0.05), but not for erythema, edema, and rash (*p* > 0.05). Pairwise age-group comparisons demonstrated differences between age groups in softness, smoothness, overall skin condition, dryness, and scaling; again, differences in dryness and scaling were likely to be driven by the slightly higher scores in the 3- and 5-year-old groups (Fig. 1). Overall, the clinical data from the assessments of the children’s skin indicated a general increase in score with age in softness, smoothness, and overall skin condition.

### Instrumental evaluations

TEWL measured on the cheek was greater in 1-year-old children when compared with children older than age 5 or adults (Fig. 2a).This finding was consistent with a more permeable skin barrier in the younger children.

Skin surface moisture, as measured by capacitance (Corneometer), was generally not influenced by children’s age on the cheek; however, on the ventral forearm, capacitance at age 10 was reduced compared with ages 1 and 2 (Fig. 2b). Corneometer measurement of skin moisture was significantly greater in mothers than in children in all age groups on the cheek, and it was greater on the ventral forearm at all ages older than 1 (Fig. 2b).

Skin moisture, as measured by high-frequency conductance (Skicon) on the ventral forearm, decreased significantly and differed between age groups in children, from a mean value of 78 in 1 year to 35 in 10 years (ANOVA, *p* < 0.001; Fig. 2c). Pairwise comparisons showed significantly higher conductance in 1 year versus 3-, 4-, and 10 years, suggesting a trend for a decrease in baseline skin conductance at this site with age. This decrease in forearm skin moisture was consistent with Corneometer measurements at the same site. No consistent difference between mothers and children was observed in forearm conductance; however, mothers had greater conductance than 4- and 10 years.

Water-holding capacity was monitored by measuring the uptake (sorption) and release (desorption) of water from the skin. Water sorption and desorption profiles were obtained from children and their mothers on the ventral forearm immediately after baseline using high-frequency conductance. Values in 1 year and their mothers at baseline and at 0, 30, 60, and 120 s are shown in Fig. 2d, where it was found that there was a higher water-holding trend in children than adults. Desorption values in 2 years were lower compared with those aged 3 and 10 years (Fig. 2e), indicating greater tendency of the skin to hold water.

**Fig. 3:**
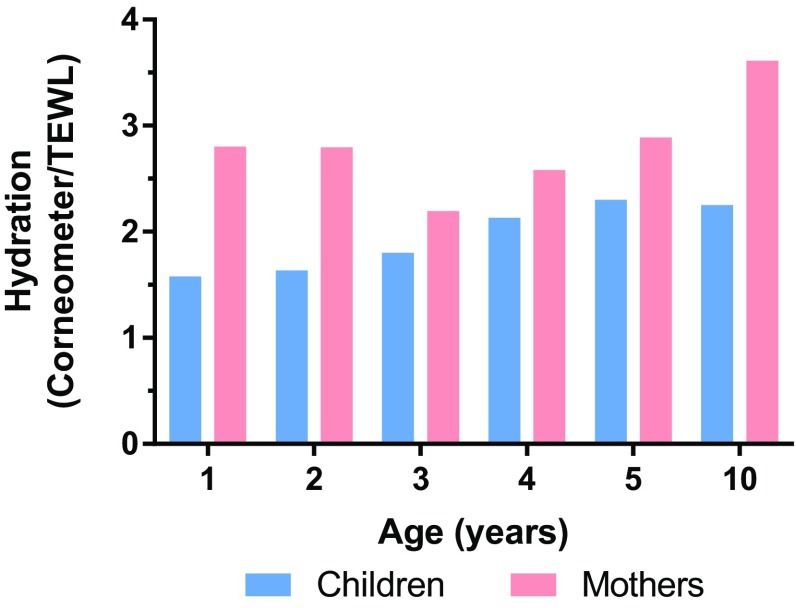
Skin hydration varies with age. Hydration calculated as ratio of Corneometer reading over TEWL value. *TEWL* transepidermal water loss

Overall skin hydration can be considered as a ratio of skin moisture (water content) to water loss. The ratio of water content (as measured by Corneometer) to TEWL (as measured by Vapometer) suggested that overall skin hydration in children increased through age 5, at which point it began to approach the adult value (i.e., the ratio observed in mothers; Fig. 3).

## Discussion

This study demonstrated significant changes with age in skin characteristics (measured clinically and instrumentally) of Chinese children. The clinical evaluations indicated a general decrease in softness, smoothness, and overall skin condition (measured as increased scores) with children’s age. Although our study was performed in a homogeneous ethnic group, our findings were consistent with those in several different ethnic groups [4]. As children age, there are significant decreases in TEWL and a concomitant decrease in moisture content, plateauing at about 5 years of age [11]. The scores in children versus adults seen in this study are consistent with a maturing of the skin barrier over time [4, 11].

Unexpectedly, the 3 years had the highest scores for dermatology assessments. The reason for the differences is not clear, and could not be explained by a difference where the child was born (Shanghai versus other locations). The mean scores in this group were still within the very good range (<2), meaning that although slightly higher, they were not considered out of the ordinary or abnormal. Individuals were not excluded from study entry unless the characteristics were clinically determined to be moderate to severe (scores >7).

The water sorption–desorption test can be used to provide information about the water-holding capacity of the skin over a short time scale [10]. One-year-old children had slightly greater sorption than their mothers in this study. This finding is consistent with a more permeable skin barrier to water in younger children than in adults [4, 5, 11], although the difference has not been observed by all investigators [3].

Two different measures (Corneometer, Skicon) were used to evaluate possible changes in skin moisture content with age on the ventral forearm. Both methods showed similar results, reinforcing the concept that moisture content at this site was highest in 1 year and decreased with age (however, on the cheek, no consistent change was observed by age). The finding that the children’s mothers had greater skin moisture content than the children contrasted with findings from others [4] who demonstrated moisture content of adults was lower than infants/toddlers. These differences may perhaps have been due to previous moisturizer use by the mothers or to differences in technique and/or site of measurement. Overall hydration, measured as the ratio of Corneometer value to TEWL [12], increased with age, and suggested that the maturation of the skin barrier (e.g., reduced TEWL) had a greater effect on skin moisture than the smaller decrease in Corneometer or Skicon value and that the overall effect was to increase skin hydration.

This was a cross-sectional study performed over a short period of time and not a longitudinal study. We cannot assume that changes observed in one of the studied age groups will progress as these specific children age. In addition, each group of subjects was relatively small (≤30 individuals) making generalizations to other populations difficult. Because the age cohorts were small, we did not make any attempt to analyze the data stratified by sex and, therefore, were unable to determine whether sex influenced skin properties subjects studied.

In summary, this study used both clinical and instrumental methods to assess skin condition and skin barrier function in children and adults. Clinically, skin was generally perceived to be softer and smoother and in better overall condition in younger children. As expected with skin maturation, the clinical scores tended to increase with age.
